# Contribution of Mössbauer spectroscopy to the investigation of Fe/S biogenesis

**DOI:** 10.1007/s00775-018-1534-z

**Published:** 2018-01-19

**Authors:** Ricardo Garcia-Serres, Martin Clémancey, Jean-Marc Latour, Geneviève Blondin

**Affiliations:** 1 Univ. Grenoble Alpes, CEA, CNRS, LCBM UMR 5249, pmb, 38000 Grenoble, France; 2 LCBM/pmb, CEA Bât C5, 17 Rue des Martyrs, 38054 Grenoble Cedex 9, France

**Keywords:** Mössbauer spectroscopy, Fe/S biogenesis, Iron trafficking, Iron–sulfur cluster

## Abstract

**Abstract:**

Fe/S cluster biogenesis involves a complex machinery comprising several mitochondrial and cytosolic proteins. Fe/S cluster biosynthesis is closely intertwined with iron trafficking in the cell. Defects in Fe/S cluster elaboration result in severe diseases such as Friedreich ataxia. Deciphering this machinery is a challenge for the scientific community. Because iron is a key player, ^57^Fe-Mössbauer spectroscopy is especially appropriate for the characterization of Fe species and monitoring the iron distribution. This minireview intends to illustrate how Mössbauer spectroscopy contributes to unravel steps in Fe/S cluster biogenesis. Studies were performed on isolated proteins that may be present in multiple protein complexes. Since a few decades, Mössbauer spectroscopy was also performed on whole cells or on isolated compartments such as mitochondria and vacuoles, affording an overview of the iron trafficking.

**Graphical abstract:**

This minireview aims at presenting selected applications of ^57^Fe-Mössbauer spectroscopy to Fe/S cluster biogenesis
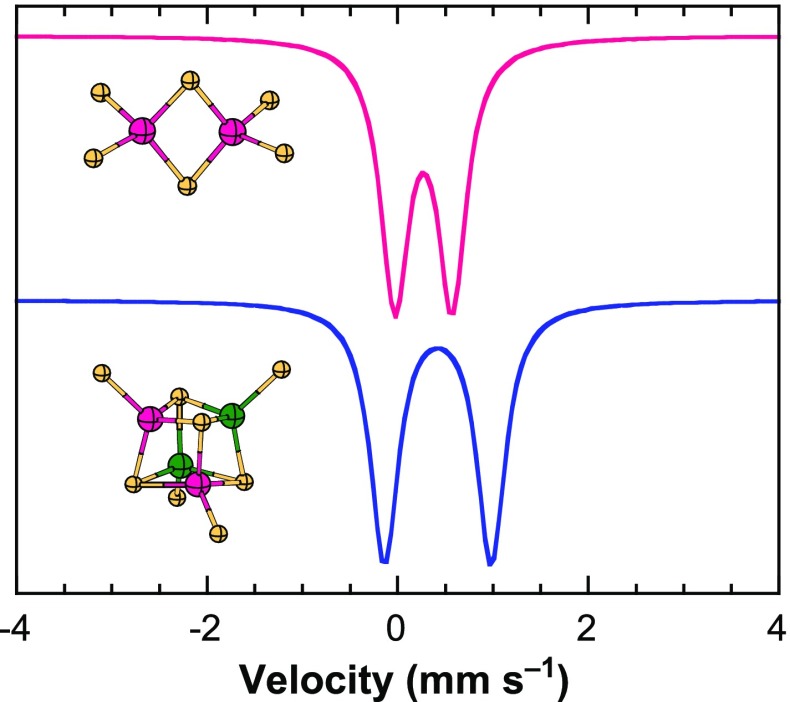

## Introduction

Mössbauer spectroscopy is a powerful probe for the investigation of proteins and enzymes with iron active sites. Accordingly, it has been used extensively for the study of Fe/S clusters [1–3]. This spectroscopy relies on the resonant absorption of γ photons by ^57^Fe nuclei [4, 5]. Mössbauer spectroscopy allows the determination of the nuclearity of the clusters, the identification of oxidation states of the iron ions, and provides information on their coordination spheres. It also reveals the spin states of the iron sites, and their detailed electronic structures (magnetic couplings and electron delocalization) can be established [6–8]. Another main advantage of this spectroscopy resides in its capability to quantify the different forms of iron within a sample, the absorption of each type of iron ions being proportional to its concentration. For all these reasons, Mössbauer spectroscopy is perfectly suited to investigate the iron–sulfur cluster assembly machinery.

Because the ^57^Fe isotope is present at only 2% in natural abundance, full enrichment is often required when investigating proteins. Isolated proteins can be purified as-is or reconstituted with a ^57^Fe source. When purified as-is, the cells are grown on minimal media, and then provided abundant ^57^Fe at the time of induction of the overexpressed genes. Purification must be done anaerobically, to avoid air oxidation of Fe/S clusters. Alternatively, the apo protein can be purified aerobically and then reconstituted anaerobically with a ^57^Fe salt. Reconstitution of Fe/S clusters makes the purification process easier, and sometimes the proteins can simply not be purified with their clusters.

This review aims at presenting a selection of results relevant to Fe/S biogenesis, where Mössbauer spectroscopy was crucial in elucidating or at least improving the knowledge of the mechanism of Fe/S cluster assembly. In vitro studies will be first described, dealing either with single proteins or with multiple protein complexes. Possible protein–protein interactions in vivo may lead to subtle changes in the Fe/S cluster environments, resulting in variations between what is observed in vitro and what is in fact present in the cells. This was highlighted in the case of a SAM-dependent enzyme in a 2005 paper by Broderick and co-workers [9]. As a consequence, Mössbauer studies performed on whole cells are very informative and some recent works will be presented in this minireview.

## Evidencing histidine coordination

**Fig. 1:**
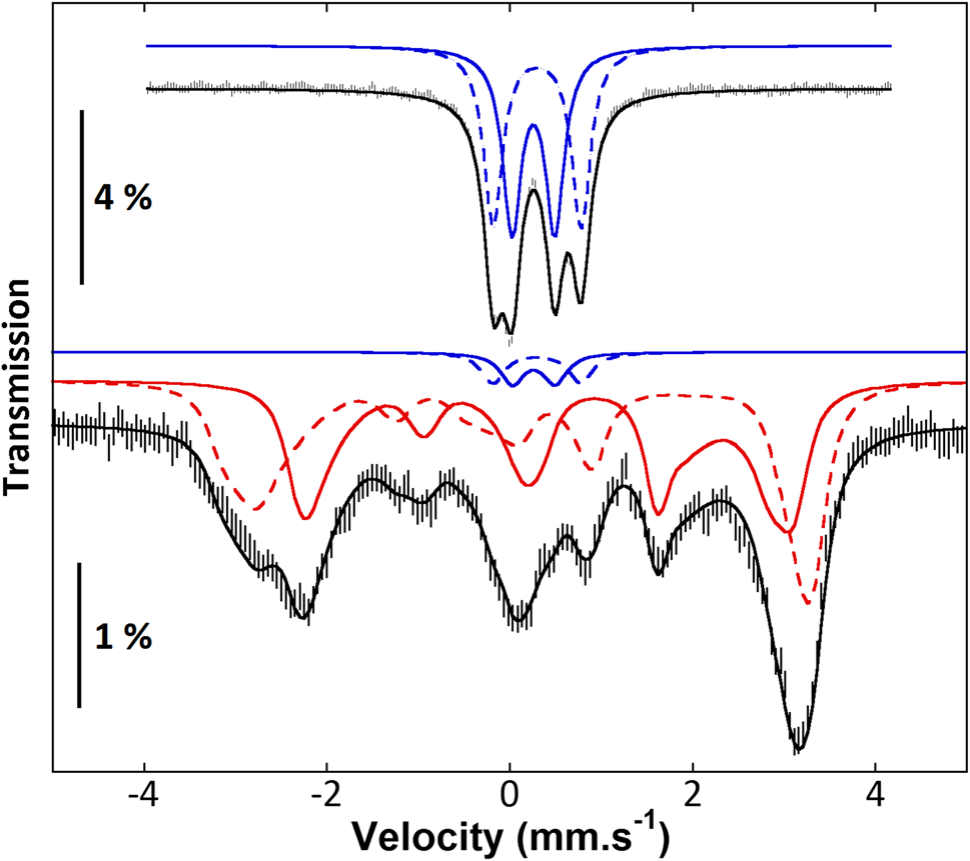
Characterization by Mössbauer spectroscopy of oxidized (top) and dithionite-reduced (bottom) human mitoNEET recorded at 4.2 K in a magnetic field of 600 G applied parallel to the direction of the γ-rays. The solid and dashed blue lines represent the contributions of [2Fe-2S]^2+^ clusters, and the solid and dashed red lines represent the contributions of [2Fe-2S]^+^ clusters. From [15]

Iron sites in Fe/S clusters are generally tetrahedral with four sulfur-coordinating atoms. These can be the thiolate function of cysteine side chains or bridging sulfide ions. However, there are exceptions. In a 2012 study by Münck and co-workers [10], Mössbauer was instrumental in characterizing the [2Fe-2S]^+^ cluster of the IscR promoter as being ligated by three cysteines and one histidine, with the histidine bound to the ferrous center. IscR has been found to promote Fe/S cluster biosynthesis through the Isc pathway in aerobic conditions [11]. The (Cys)_3_(His)_1_ ligation for [2Fe-2S] clusters has been linked to a role for the proteins as redox sensors [12–14]: In the oxidized form, the cluster is less tightly bound, allowing for transfer to other proteins of the machinery. This is the case for the mammalian outer mitochondrial membrane protein mitoNEET, which plays a role in Fe-S cluster repair, where cluster transfer is triggered by oxidative stress conditions [15]. The oxidized and reduced forms of mitoNEET are readily distinguished by their Mössbauer spectra (Fig. 1) [15]. While the diamagnetic, oxidized [2Fe-2S]^2+^ form yields two quadrupole doublets (blue lines in Fig. 1), the *S* = 1/2, reduced [2Fe-2S]^+^ form is split by Zeeman effect on nuclear spin states (red lines in Fig. 1).

In the oxidized form, the cluster yields two inequivalent doublets in a 1:1 ratio, each one corresponding to one iron center, with isomer shifts of 0.26 and 0.30 mm/s. The difference of 0.04 mm/s is evidence of the different coordination environments, the coordinated histidine favoring a higher isomer shift compared to cysteine coordination. As a comparison, the Mössbauer spectrum of mammalian ferrochelatase, which has a [2Fe-2S]^2+^ cluster with (Cys)_4_ coordination, is a perfectly symmetric doublet [16], while the two iron sites of the [2Fe-2S]^2+^ cluster of Rieske proteins, with (Cys)_2_(His)_2_ ligation, have a difference in isomer shifts of 0.08 mm/s [17]. A difference of 0.05 mm/s, similar to IscR and mitoNEET, is observed for the scaffold protein IscU in *E. coli*, which can bind a [2Fe-2S]^2+^ cluster coordinated by three cysteines and a solvent-exposed coordination site [18]. In the reduced [2Fe-2S]^+^ form, the Mössbauer spectrum analyzed with an *S* = 1/2 spin Hamiltonian (Fig. 1, red lines) yields for the Fe(II) site an isomer shift that is a decreasing function of the number of coordinated cysteines [15]: 0.62 mm/s for *A. aeolicus* FdI (2 cysteines), ~ 0.70 mm/s for human mitoNEET or *E. coli* IscR (1 cysteine and 1 histidine), and 0.75 mm/s for Rieske proteins (2 histidines).

In other instances, a cysteine to histidine change in the coordination of [2Fe-2S]^2+^ clusters impacts the function of proteins directly related to the iron trafficking in the cell. For example, it seems to play a role in controlling the location of the Aft1 transcription factor. Under iron-replete conditions, Aft1 is located in the cytosol. Under iron-depleted conditions that may originate from a defect in mitochondrial Fe/S biogenesis, Aft1 moves to the nucleus and activates iron regulon genes [19]. The two Fe repressors of activation, Fra1 and Fra2, and the two glutaredoxins Grx3 and Grx4 have been identified as key proteins in this process [20–22]. Both Grx3 and Grx4 form homodimers that are bridged by a [2Fe-2S]^2+^ cluster. Mössbauer spectroscopy clearly established full cysteine coordination, two from the CGFS active site of Grx3/4, and two from reduced glutathione (GHS) molecules [23]. Similar coordinations have been observed in other glutaredoxins [24, 25]. It was demonstrated that the Grx3/4 homodimers were unable to regulate Aft1 when lacking the [2Fe-2S] cluster [26]. Moreover, both Grx3 and Grx4 strongly interact with Fra2, as substantiated by the isolation of Fra2/Grx3 and Fra2/Grx4 heterodimers. In these heterodimers, the bound [2Fe-2S]^2+^ is coordinated by a single histidine, as evidenced by Mössbauer spectroscopy and Q-band ENDOR [23]. The histidine has later been identified as histidine 103 from Fra2 [27]. A similar heterodimer has been evidenced with the human Grx3 and BolA2 proteins [28], the latter being the closest human analogue of the yeast Fra2 protein. The C-terminal Grx-domain of Grx3 was demonstrated to be critical for both the interaction of Grx3 with Fra2 and for the inhibition of Aft1 activity in vivo. This has led proposing the [2Fe-2S] Fra2-Grx3/4 complex as a candidate for Aft1 regulation and as a novel Fe/S cluster binding regulatory complex [29].

## [2Fe-2S] and [4Fe-4S] clusters: conversions and transfers to apo proteins

**Fig. 2:**
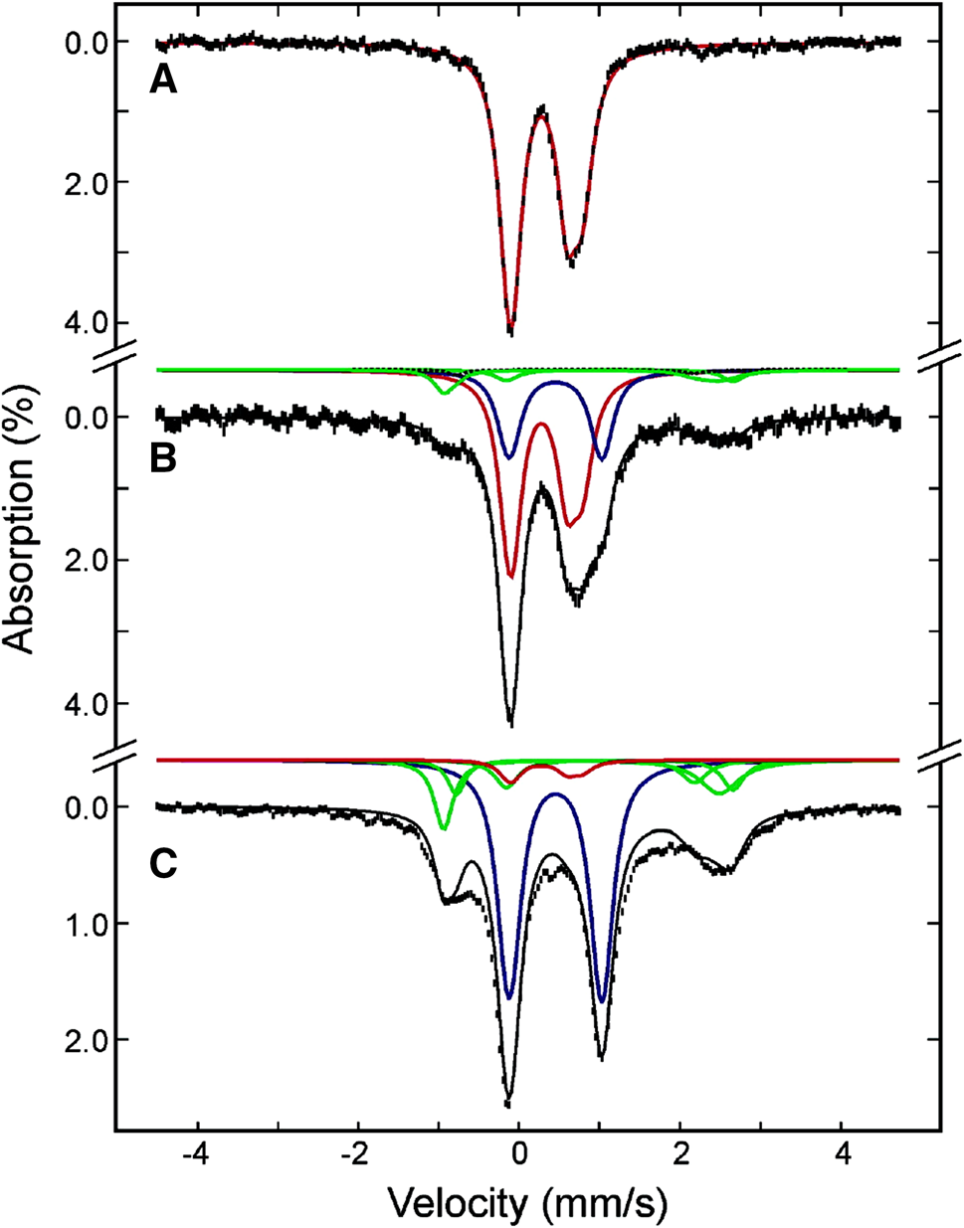
Ferredoxin-mediated reductive coupling of [2Fe-2S]^2+^ clusters on IscU monitored by Mössbauer spectroscopy (4.2 K; 50 mT applied field parallel to γ radiation). 2 × [2Fe-2S]^2+^ IscU as prepared (**a**), after reduction with 1.06 reducing equivalents of Fdx [2Fe-2S]^2+^ cluster (**b**), and after reduction with 1.28 reducing equivalents of dithionite per IscU [2Fe-2S]^2+^ cluster (**c**). From [18]

Mössbauer spectroscopy allows the discrimination between [2Fe-2S] and [4Fe-4S] clusters, even when both are in the diamagnetic forms with a 2+ charge. Huynh and co-workers have used Mössbauer spectroscopy to monitor the NifS-mediated assembly of Fe/S clusters on NifU [30], and the IscS-mediated fixation of Fe/S clusters on IscU [31]. In the latter study, they have evidenced the sequential fixation of one [2Fe-2S]^2+^ cluster per IscU dimer, then two [2Fe-2S]^2+^ clusters per IscU dimer, then one [4Fe-4S]^2+^ cluster per IscU dimer. Subsequently, by isolating the fraction that contained two [2Fe-2S]^2+^ clusters per IscU dimer, they proved that dithionite is able to reductively couple the two [2Fe-2S]^2+^ clusters into one [4Fe-4S]^2+^ cluster, and that reduced Isc Ferredoxin is also a capable reductant for this purpose [18]. An added difficulty in the last experiment is that, in the process of reductive coupling, the [2Fe-2S]^+^ cluster of reduced IscFdx is oxidized into a [2Fe-2S]^2+^ cluster, the signal of which interferes with that from IscU–[2Fe-2S]^2+^. Mössbauer spectroscopy comes in particularly handy here: Since only the ^57^Fe isotope is detected, it is possible to “light up” a given fraction of the iron ions using ^57^Fe-enriched iron, and “switch off” another fraction using natural iron for that fraction. Owing to the low natural abundance of ^57^Fe, the non-enriched fraction is effectively Mössbauer-silent when mixed with a comparable amount of enriched iron. In this case, Huynh and co-workers used ^57^Fe-enriched IscU and non-enriched IscFdx. Before reduction (Fig. 2a), the spectrum shows the exclusive signal of 2 × [2Fe-2S]^2+^ IscU. After addition of 1.06 reducing equivalents of IscFdx (Fig. 2b) the signal of [4Fe-4S]^2+^ IscU is detected (blue line), but 61% of the initial [2Fe-2S]^2+^ stay unreacted (red line). By contrast, dithionite reduction (Fig. 2c) leads to 77% transformation to [4Fe-4S]^2+^ and barely any unreacted [2Fe-2S]^2+^. The difference is attributed to the redox potential of IscFdx, which is higher than that of dithionite, and would be similar to the apparent redox potential of the reductive coupling. This is interpreted by the authors as a way for the Isc machinery to modulate the balance between the [2Fe-2S]^2+^ and [4Fe-4S]^2+^ cluster-bound forms of IscU.

In an accompanying paper [32], the same authors showed that only the [4Fe-4S]-loaded form of IscU is competent for the activation of *A. vinelandii* apo-aconitase. Because it was previously demonstrated that holo-Fdx can be formed from [2Fe-2S]-IscU and apo-Fdx [33], they propose that cluster binding on IscU induces conformational changes that are responsible for the specific interaction of [4Fe-4S]-IscU with target [4Fe-4S] proteins and [2Fe-2S]-IscU with target [2Fe-2S] proteins. More recently, a similar flexibility was reported for *Arabidopsis thaliana* Nfu2 [34]. Although the studies are very convincing, direct transfer of an intact [4Fe-4S] cluster from holo-IscU to target proteins has so far only been proved for *A. vinelandii*, and there is no consensus on the fact that [4Fe-4S]-IscU may be the physiologically competent form of IscU. On the contrary, several clues point toward the 1 × [2Fe-2S]/dimer as the physiological form of IscU proteins [35]. Finally, a species-dependent variability of behaviors cannot be discarded.

Aside from U-type proteins, the role of A-type proteins has also been investigated using Mössbauer spectroscopy. A-type proteins are expressed from the same operon as their U-type counterparts. But whereas U-type proteins have been proved to act as scaffolds in Fe/S biosynthesis [36, 37], the true role of A-type proteins is largely under debate. It has been evidenced with the help of Mössbauer spectroscopy that A-type proteins in vitro are able to bind [2Fe-2S] clusters as well as [4Fe-4S] clusters, hence a hypothesized role as alternative Fe/S scaffolds. However, this role was challenged by in vitro studies [38, 39]. Another proposed role is that of primary iron donors (to which frataxin is also a candidate). Mononuclear Fe(III) and Fe(II) complexes of *Azotobacter vinelandii* ^Nif^IscA have been thoroughly characterized by Mössbauer, EPR and MCD spectroscopies [40]. Spin Hamiltonian parameters of the Fe(II) form are very similar to those of reduced rubredoxins, whereas the Fe(III) form displays an unprecedented *S* = 3/2 rhombic intermediate-spin ground state, indicating a five-coordinate ligation with two or three cysteinate ligands and respectively three or two non-cysteinate ligands. Both the Fe(III) and Fe(II) forms are able to release Fe(II) in the presence of l-cysteine, but no direct transfer of iron to NifU-1 could be evidenced. Hence, a role as a specific iron donor is not a favored hypothesis. Other proposed roles for A-type proteins are: Downstream carriers for clusters assembled on U-type proteins, or metallochaperones that facilitate in situ assembly of [4Fe-4S] clusters on target proteins from [2Fe-2S] clusters assembled on U-type proteins. In a paper published back to back with the one mentioned above, the authors show cycling between a form of ^Nif^IscA containing one [2Fe-2S]^2+^ cluster per homodimer, and one containing one [4Fe-4S]^2+^ cluster per homodimer [41]. [2Fe-2S]^2+^ to [4Fe-4S]^2+^ conversion is induced by DTT addition, and [4Fe-4S]^2+^ to [2Fe-2S]^2+^ conversion is induced by O_2_ exposure. The authors conclude that ^Nif^IscA may be able to directly transfer a [4Fe-4S] to target proteins in anaerobic conditions, whereas in aerobic conditions both the Fe(III)-bound and the [2Fe-2S]^2+^-bound forms may play a role in Fe/S protein maturation. This supports the findings made on other A-type proteins such as ErcA, which is able to assemble either a [2Fe-2S] or a [4Fe-4S] cluster, and transfer a [4Fe-4S]^2+^ cluster to IspG, an iron–sulfur enzyme requiring ErpA function in vivo [42]. After incubation of [4^57^Fe–4S] ErpA with apo IspG and subsequent purification, the Mössbauer spectrum of the purified IspG displayed the characteristic features of [4Fe-4S]^2+^ clusters.

As mentioned above, reaction with molecular oxygen may be deleterious for [4Fe-4S] clusters. It has been observed in both the mitochondrial and cytosolic iron–sulfur cluster assembly machineries, the latter being far less well understood than the former. The yeast Dre2 protein and the human anamorsin (also called CIAPIN1) analogue are suspected to play a critical role in the cytosolic Fe/S biogenesis. Two binding domains have been identified, each one containing four cysteines. There is a debate on the number and nature of the accommodated Fe/S clusters. In combination with other techniques, Mössbauer spectroscopy allowed to propose a unifying view [43]. When Dre2 is isolated under anaerobic conditions, a [2Fe-2S]^2+^ is observed in the N-terminal domain, whereas a [4Fe-4S]^2+^ cluster is present in the C-terminal domain. When isolated under oxidative conditions, the [4Fe-4S]^2+^ cluster is partially converted into a [2Fe-2S]^2+^. The same study suggests that the N- and C-terminal clusters are concertedly assembled. Further investigations are required to determine the evolution of the Dre2 Fe/S clusters.

As highlighted above, the fact that a protein is able to assemble and transfer a [4Fe-4S] cluster in vitro, does not mean that it is an iron–sulfur scaffold in vivo. To sort between biologically relevant species and experimental artifacts, specific experiments have to be designed. In some instances, the common practice of reconstituting proteins with ^57^Fe salts can be replaced by direct purification of ^57^Fe-enriched proteins. This requires growing the cells in Fe-minimal media, and then providing a ^57^Fe salt at the time of induction of the overexpressed gene. This technique has been used to record the Mössbauer spectrum of ^57^Fe-labeled SufA purified from the SufABCDSE expression system [44]. Coexpression of the whole operon was necessary because of the tight interplay between proteins in the Fe/S cluster assembling process. The study revealed that as-purified SufA contains a [2Fe-2S]^2+^ cluster with complete cysteinyl ligation, which implies that the cluster is located at the dimer interface. The mechanism of formation of the Fe/S clusters in SufA was also addressed using Mössbauer spectroscopy. Thanks to the proportionality between Mössbauer absorption and ^57^Fe content, the efficiency of Fe/S cluster reconstitution can be assessed. In a study comparing the mechanism where Fe is incorporated first and the mechanism where S is incorporated first [45], it was concluded that (unlike IscA) SufA binds Fe(II) in an unspecific manner, mainly through noncysteinyl ligands, and that Fe/S cluster assembly is more efficient if sulfuration precedes iron addition.

## Multiple protein complexes

**Fig. 3:**
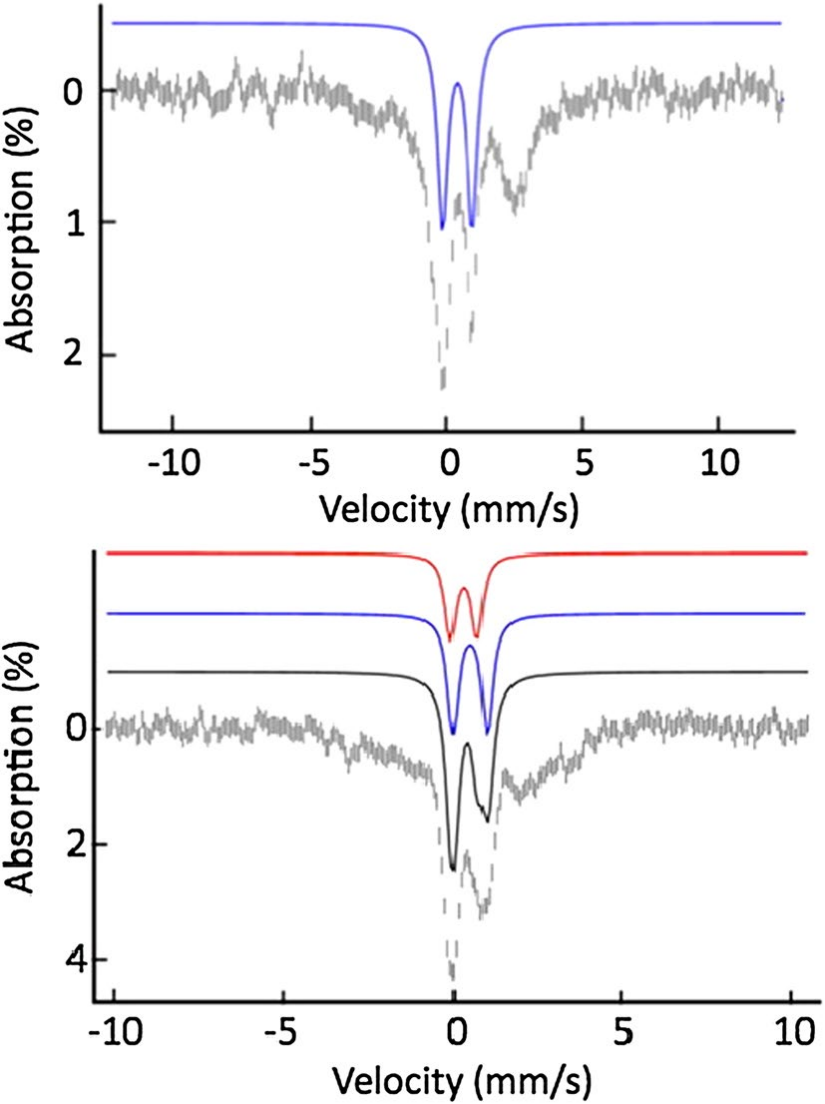
4.2 K, 60 mT parallel applied field Mössbauer spectra of the reconstituted quaternary (ISCU/NFS1/ISD11/FXN) complex (top) with 3.5 Fe/Cplx or reconstituted ternary (ISCU/NFS1/ISD11) complex (bottom) with 2.4 Fe/Cplx. The blue lines represent contributions from [4Fe-4S]^2+^ clusters and the red lines represent contributions from [2Fe-2S]^2+^ clusters. Adapted from [47]

Another method for studying the interplay between proteins of the same operon is to purify them together, and then add ^57^Fe(II), l-cysteine and cysteine desulfurase to reconstitute Fe/S clusters in protein complexes. This has been done, for instance with the SufBC_2_D complex, which is able to assemble a [4Fe-4S]^2+^ cluster displaying in Mössbauer a symmetrical quadrupole doublet with identical parameters to the cluster assembled on SufB only [46]. It was, therefore, postulated that the species that is competent in Fe/S cluster biosynthesis is the SufBC_2_D complex binding a [4Fe-4S]^2+^ cluster exclusively through the conserved cysteines of SufB. A similar approach was used in a 2012 study that aimed at shedding light on the role of frataxin in Fe/S cluster biosynthesis [47]. Frataxin (FXN) plays an important role in Fe/S cluster biosynthesis, as evidenced by the fact that the Fe/S cluster biosynthesis machinery is disrupted when frataxin is deleted. Although the precise role of frataxin is unknown, it has been proposed as a primary iron transporter. In this study involving murine proteins, it was proved to stabilize the ternary (ISCU/NFS1/ISD11) complex. The Mössbauer spectra of the reconstituted quaternary (ISCU/NFS1/ISD11/FXN) and ternary (ISCU/NFS1/ISD11) complexes proved unambiguously that both complexes were able to assemble a [4Fe-4S]^2+^ cluster. However, both the total iron content per complex and the proportion of iron in the form of [4Fe-4S]^2+^ clusters were higher for the quaternary complex (Fig. 3). Additionally, the quaternary complex was found to be 3 times more efficient than the ternary complex in transferring its cluster to mitochondrial aconitase, hinting to a double role of frataxin in iron import and Fe/S complex export.

In a recent study, these results have been questioned [48, 49], as frataxin was proposed to accelerate a rate-limiting transfer step in the formation of [2Fe-2S] clusters. It was also suggested that a competitive Fe/S mineralization occurs in the cytosol.

## Studies on whole cells with a single-induced protein

The first whole-cell Mössbauer studies were performed using a labelled ^57^Fe-siderophore for iron uptake [50–52]. More recently, studies on cells with a single overexpressed protein participating in Fe/S cluster assembly were reported. These experiments are usually performed by overexpressing the protein in *E. coli*. Identification of the Fe/S cluster form of the protein of interest is performed by comparing the spectra recorded on cells with and without the induced protein.

**Fig. 4:**
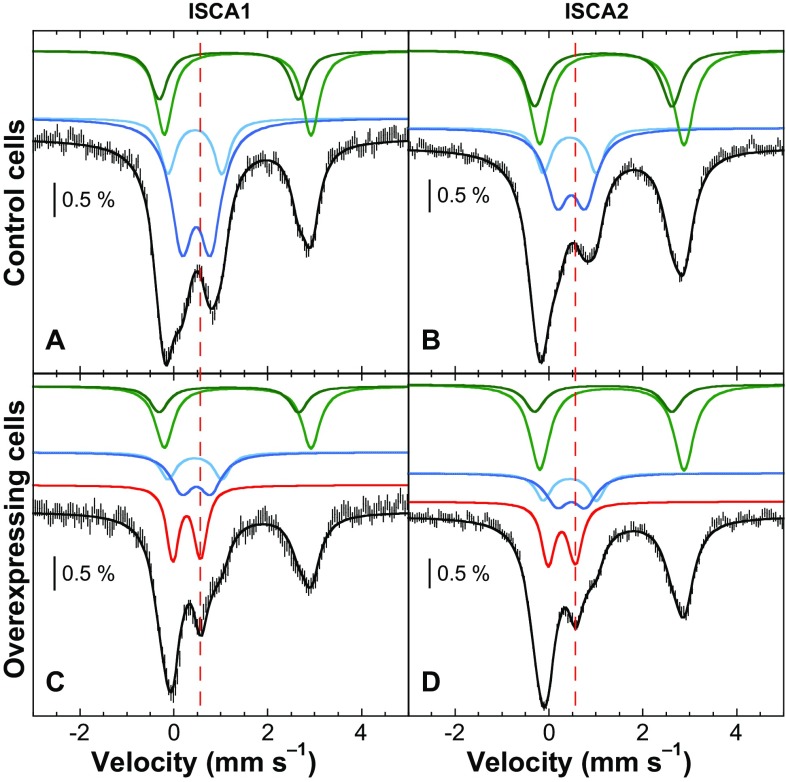
Mössbauer spectra recorded at 5.5 K using a 60 mT external magnetic field applied parallel to the γ-beam. The panels **a** and **b** reproduce spectra of whole control cells and panels **c** and **d** those of ISCA1 or ISCA2-overexpressing cells. Experimental spectra are shown with hatched marks and simulations are overlaid as solid black lines. Five components were used for simulation: HS Fe^II^ (light and dark green), [4Fe-4S]^2+^ clusters and LS ferrous hemes (light blue), Fe^III^ NP (dark blue) and [2Fe-2S]^2+^ (red). From [55]

Typical spectra recorded at low temperature in a weak external magnetic field on *E. coli* cells lacking the overexpressed protein are shown in Fig. 4 and have been observed in several studies [9, 52–54]. Such spectra extend from − 1 to + 3.5 mm/s. The simulations and the associated iron content will be described below.

One of the first whole-cell Mössbauer studies related to Fe/S biogenesis involved the transcription factor IscR [11]. The low-temperature low-field Mössbauer spectra recorded on the anaerobically isolated protein revealed a mixture of the oxidized and reduced forms. Measurements were thus performed on whole cells to determine the physiological form of the cluster in vivo [10]. The spectrum recorded at 4.2 K in a 45 mT external magnetic field on cells with overexpressed wild-type IscR extends from − 4 to + 5 mm/s, thus on a larger velocity range that the spectrum recorded under the same conditions on cells lacking IscR. This strongly suggests the presence of a magnetic cluster associated to IscR. Indeed, features on both edges of the spectrum of IscR cells evidenced the signature of the reduced *S* = 1/2 [2Fe-2S]^+^ cluster that accounts for 32% of the iron content. There was no evidence for the presence of either an oxidized [2Fe-2S]^2+^ cluster or a [4Fe-4S]^2+^ cluster. These experiments demonstrated that IscR accommodates a [2Fe-2S] cluster in vivo, which is preponderantly reduced.

The detection of the formation in vivo of a diamagnetic Fe/S cluster is more difficult because the signal is then located in the − 1/+ 1 mm/s velocity window, like the signal of the control cells. However, the iron distribution is different in cells with an overexpressed iron protein and in cells where it is not. This allows the identification of an extra signal originating from the induced protein. This situation was met for ISCA1 and ISCA2 proteins. Figure 4 reproduces the Mössbauer spectra recorded at 5.5 K with a weak external magnetic field on whole cells with or without overexpressed ISCA1/2 protein. On both induced samples, one additional absorption line is clearly detected at 0.5 mm/s, indicating the presence of a [2Fe-2S]^2+^ cluster, as determined for the isolated proteins [55]. Simulations were first performed on the two spectra of control cells. Four doublets were used. Two correspond to high-spin ferrous ions, one has nuclear parameters close to those determined for [4Fe-4S]^2+^ clusters and low-spin ferrous heme systems, and the last one corresponds to ferric nanoparticles. Once these nuclear parameters were known, simulations of the ISCA-overexpressed cell spectra were performed, allowing only the relative contributions to vary. To reduce the number of unknowns, nuclear parameters were also fixed for the additional Fe/S cluster as parameters of standard oxidized [2Fe-2S] clusters, as only these allowed the generation of satisfying simulations. Accordingly, it was concluded that both ISCA1 and ISCA2, when separately overexpressed, harbor a [2Fe-2S]^2+^ cluster in vivo. In addition, the variations of the different contributions indicate that the ferric nanoparticle pool is the principal source of iron for the generation of these Fe/S clusters.

To the best of our knowledge, no Mössbauer studies were performed on cells where more than one protein was overexpressed.

## Iron trafficking

**Fig. 5:**
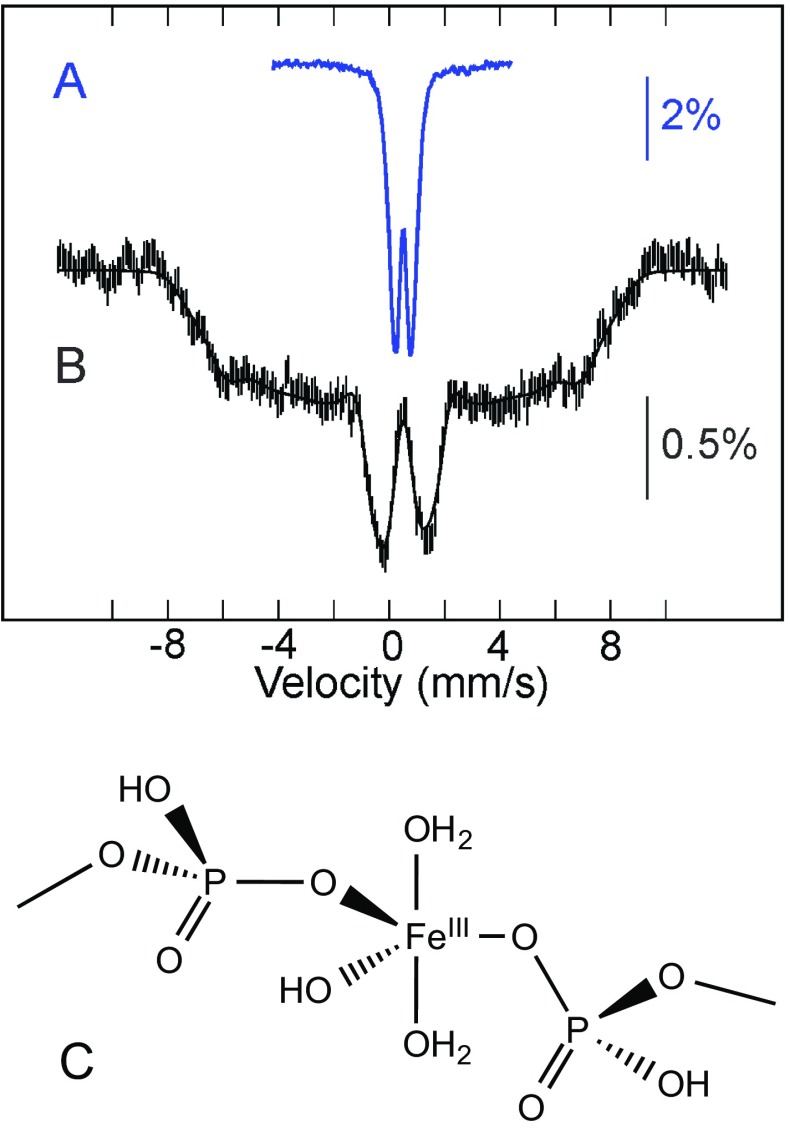
Top: Mössbauer spectra of ∆*yfh1* yeast mitochondria. Spectrum **a** was recorded at 78 K in zero field and spectrum **b** at 4.2 K in a 7 T magnetic field applied parallel to the γ-beam. Experimental spectra are shown with hatched marks and simulations are overlaid as solid lines. Simulation of spectrum **b** was achieved assuming a distribution of the hyperfine field. From [59]. Bottom (**c**): Coordination of the ferric ion in NP according to EXAFS and electron microscopy results [60]

Another way to gain more information on the Fe/S biogenesis in cells is to selectively delete genes that are involved in this process and to look with Mössbauer spectroscopy at the resulting variations in the iron distribution. This strategy has been mainly developed on the budding yeast *Saccharomyces cerevisiae*. A pioneer work was achieved by Lesuisse and coworkers on mitochondria isolated from *S. cerevisiae* lacking the *yfh1* gene [56]. Yfh1 is the yeast frataxin homologue of the human frataxin, a key protein in the elaboration of Fe/S clusters. A deficiency in frataxin has been evidenced for patients suffering from Friedreich’s ataxia, which leads to iron accumulation in their brain and heart tissues [57, 58]. Mössbauer studies on ∆*yfh1*-mutant cells showed that iron accumulates in mitochondria as amorphous ferric phosphate nanoparticles (Fig. 5). Accordingly, iron is sequestered and unavailable for Fe/S cluster and heme biogenesis.

Lindahl and coworkers have performed analogous studies on isolated mitochondria and extended them to the investigation of whole yeast cells and isolated vacuoles. As they had already described elsewhere [61], Mössbauer spectroscopy allows the identification and the quantification of groups of iron species when present at higher concentrations than 10–20 µM. Because there is no Mössbauer-silent iron form, this spectroscopy gives a reasonably good overview of the iron distribution in the investigated samples. One main drawback is the necessity of investigating cells enriched in ^57^Fe isotope, as a minimal amount of it is required to detect a Mössbauer signal. In combination with other spectroscopies and techniques, namely electron paramagnetic resonance (EPR), UV–visible, electron microscopy and inductively coupled plasma mass spectroscopy (ICP-MS), the considerable amount of data gathered for more than 10 years provided them deep insights into iron metabolism and trafficking [62, 63]. Practical aspects are detailed in references [64] and [65].

**Fig. 6:**
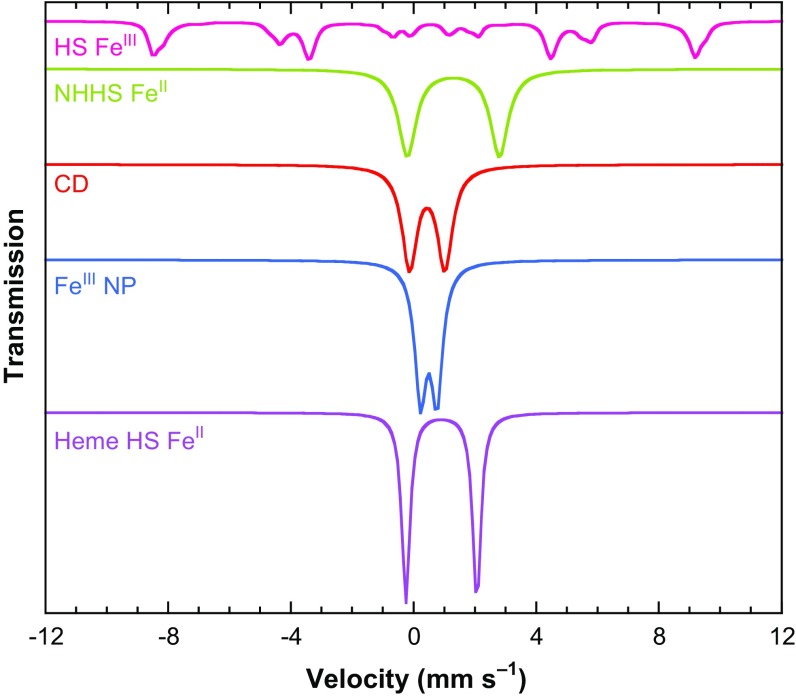
Theoretical Mössbauer spectra at 6 K with a 50 mT external magnetic field applied parallel to the γ-beam of the five main components identified in spectra recorded on *S. cerevisiae* cells, isolated mitochondria or isolated vacuoles. They are scaled to the same area and calculated according to published parameters [62, 66]

Five main groups of iron species were identified, their relative contributions depending on the sample investigated. Figure 6 displays together their low-temperature low-field Mössbauer spectra scaled to the same area. The corresponding nuclear parameters are listed in Table 1 [62].

**Table 1:** Isomer shift and quadrupole splitting values for the five iron families identified in the yeast *S. cerevisiae*

	NHHS Fe^II^	HS Fe^II^ hemes	CD	Fe^III^ NP	NHHS Fe^III^
*δ* (mm/s)	1.3	0.91	0.45	0.53	0.54
*∆E*_Q_ (mm/s)	3.0	2.31	1.15	0.45–0.63	0.39

At 5 or 6 K in a weak external magnetic field (45–60 mT), only the non-heme high-spin Fe^III^ species (NHHS Fe^III^) present a large Mössbauer spectrum that stretches from − 10 and + 10 mm/s (pink line in Fig. 6). The non-heme high-spin Fe^II^ species (NHHS Fe^II^, green line in Fig. 6) and high-spin Fe^II^ hemes (HS Fe^II^ heme, mauve line in Fig. 6) can be identified by their high-velocity lines centred close to 2.8 and 2.1 mm/s, respectively. The ferric nanoparticles (Fe^III^ NP, blue line in Fig. 6) and the central doublet (CD, red line in Fig. 6) present doublets centred at ~ 0.5 mm/s and can be discriminated upon their quadrupole splitting. The CD doublet accounts for low-spin ferrous heme species (LS Fe^II^ heme) and for diamagnetic [4Fe-4S]^2+^ clusters, as these cannot be distinguished.

Ferric nanoparticles have been detected in mitochondria [60, 67–69] and in vacuoles [66, 70]. Fe K-edge EXAFS and electron microscopy performed on mitochondria-containing NP indicate a pentacoordination by oxygen atoms for the Fe^III^ ion (Fig. 5c) [60, 69]. Depending on the batch of vacuoles, the major contribution to the Mössbauer spectra is either analogous to that of mitochondrial ferric nanoparticles or to that of magnetically noninteracting high-spin Fe^III^ ions. Based on the Mössbauer signatures of ^57^Fe polyphosphate prepared in different acetate/acetic acid-buffered media, it is proposed that pH plays a critical role [70]. All the studies performed evidenced that the accumulation of iron in the cytosol is prevented because of the potential damages that Fe^II^ ions can cause. Accordingly, it was proposed that the excess of iron is exported either into vacuoles or into mitochondria, to detoxify the cytosol [71, 72].

Non-heme high-spin Fe^II^ has been identified in mitochondria, with increased levels as the deficiency in Fe/S clusters and hemes is more pronounced. It was proposed that it serves as an iron pool for the biosynthesis thereof [68, 73]. Similar species were proposed to be present in the cytosol [72], their amount being more important under iron-deficient growth conditions [71]. Moreover, it is suspected that another non-heme high-spin Fe^II^ species is present in vacuoles of cells depleted of the vacuolar iron importer CCC1 [66].

The signatures of heme HS Fe^II^ species and [Fe_4_S_4_]^2+^ clusters are detected in mitochondria and in whole cells. Their proportion is maximized under iron-deficient conditions [71]. Conversely, the signature of [2Fe-2S]^2+^ clusters is merely detected because the associated doublet is obscured by that of the ferric nanoparticles (NP). However, they were identified in mitochondria isolated from yeast cells grown under fermenting and iron-deficient conditions [71]. The reduced form, namely [2Fe-2S]^1+^ clusters, was pinpointed in mitochondria of respiring, respiro-fermenting [73] and of iron-deficient fermenting yeast cells [71]. In addition, EPR measurements suggested the presence of [3Fe-4S] clusters in mitochondria. However, their amount is low, precluding their detection by Mössbauer spectroscopy [64].

## Concluding remarks

We have presented here examples of the use of Mössbauer spectroscopy in the field of Fe/S biogenesis. There are many others, which we have not presented, like the stabilization of [2Fe-2S] clusters by full glutathione coordination [74]. Such species are suspected to transiently store Fe/S clusters and regulate Fe/S biosynthesis [75]. The different pathways of Fe/S assembly all involve iron in different states along the process. For this reason, Mössbauer spectroscopy is one of the best-suited techniques. It is well appropriate in conjunction with other techniques, such as EPR or resonance Raman. The advantages of Mössbauer spectroscopy are that it detects all iron in a sample, regardless of physical state, spin or oxidation state, and that the signal is proportional to the amount of iron in the sample. Other advantages are that it is measured in bulk on frozen solutions, so samples are not subject to degradation, and that it is rather inexpensive, compared, for example, to synchrotron-based techniques. Although it is a nuclear spectroscopy based on gamma-ray resonant absorption, the gamma-rays are produced by the decay of a parent isotope, which can be packed in a pocket-size source. Therefore, Mössbauer spectrometers require no large-size installation. Among all Mössbauer-active isotopes, ^57^Fe is by far the best suited for spectroscopy, which is very fortunate for biologists, and in particular for researchers in the field of Fe/S clusters. Let us conclude with a quote from a prominent scientist in the field: “You have to do Mössbauer. If you are not doing Mössbauer, you are making mistakes” (M. K. Johnson, 2007).

## Abbreviations

SAM: S-adenosyl-l-methionine
GSH: Glutathione
ENDOR: Electron-nuclear double resonance
EPR: Electron paramagnetic resonance
MCD: Magnetic circular dichroism
DTT: Dithiothreitol
ICP-MS: Inductively coupled plasma mass spectrometry
