# Correction to: Contribution of Mössbauer spectroscopy to the investigation of Fe/S biogenesis

**DOI:** 10.1007/s00775-018-1572-6

**Published:** 2018-06-02

**Authors:** Ricardo Garcia-Serres, Martin Clémancey, Jean-Marc Latour, Geneviève Blondin

**Affiliations:** 1 Univ. Grenoble Alpes, CEA, CNRS, LCBM UMR 5249, pmb, 38000 Grenoble, France; 2 LCBM/pmb, CEA Bât C5, 17 Rue des Martyrs, 38054 Grenoble Cedex 9, France

## Correction to: JBIC Journal of Biological Inorganic Chemistry 10.1007/s00775-018-1534-z

The article “Contribution of Mössbauer spectroscopy to the investigation of Fe/S biogenesis”, written by Ricardo Garcia‑Serres, Martin Clémancey, Jean‑Marc Latour, Geneviève Blondin was originally published electronically on the publisher’s internet portal (currently SpringerLink) without open access.

The copyright of the article changed on 28, May to © The Author(s) 2018 and the article is forthwith distributed under the terms of the Creative Commons Attribution 4.0 International License (http://creativecommons.org/licenses/by/4.0/), which permits use, duplication, adaptation, distribution and reproduction in any medium or format, as long as you give appropriate credit to the original author(s) and the source, provide a link to the Creative Commons license and indicate if changes were made.

The original article has been corrected.

